# Reverse Translational Approach Using Biomaterials and Stem Cells for Intervertebral Disc Degeneration

**DOI:** 10.31662/jmaj.2024-0048

**Published:** 2024-06-10

**Authors:** Katsuhisa Yamada, Hideki Sudo, Norimasa Iwasaki

**Affiliations:** 1 Department of Orthopedic Surgery, Hokkaido University Hospital, Sapporo, Japan

**Keywords:** intervertebral disc degeneration, low back pain, soft biomaterial, mesenchymal stem cell

Low back pain is a major health problem that interferes with daily life. Intervertebral disc (IVD) degeneration is a major cause of low back pain. Although analgesics and physical therapy are commonly used to manage chronic low back pain associated with IVD degeneration, neither of these approaches directly addresses the etiology of symptoms ^(1), (2)^. Surgical treatment for spinal disorders can also provide benefits. However, a treatment that can control IVD degeneration itself has not yet been developed, and these limitations have drawn attention to the potential of alternative therapies ^(1), (2)^. Recently developed tissue engineering approaches have revealed the molecular cascade involved in the degeneration of IVDs, and regenerative therapies using cells and biomaterials have gained attention as treatments aimed at regenerating degenerated IVDs ^(1), (2), (3)^.

The IVD consists of nucleus pulposus (NP) and annulus fibrosus. NP is a gelatinous tissue that contains 70%-90% of water; it is rich in extracellular matrix but low in cell density ^(1)^. IVD degeneration is characterized by loss of water content and extracellular matrix degradation, and it is considered that degenerated IVDs do not spontaneously regenerate due to poor nutrient supply and low cell mitotic capacity ^(4), (5)^. Thus, the development of therapies aimed at regenerating NP could help improve the function of IVDs ^(5)^.

As a development approach to the next-generation treatment for IVD disorders, the possibility of regulating IVD degeneration from the biomechanical and molecular biological aspects of IVD degeneration has been demonstrated (Figure 1, left) ^(1)^. Because IVD cell apoptosis is a characteristic phenomenon that occurs in the early stages of IVD degeneration ^(1), (6)^, it has been demonstrated that genetically controlling IVD cell apoptosis may be beneficial in IVD tissue degeneration ^(4), (5), (6), (7)^. As a biomaterial for tissue repair, the administration of a highly purified alginate-based hardening gel (UPAL) alone (cell-free) after discectomy was found to optimize the tissue repair environment *in vivo* and promote the self-repair ability of the residual tissue, resulting in the spontaneous repair of the IVD (Figure 1, left) ^(8)^. An *in vivo* study of rabbits implanted with UPAL hydrogel after IVD aspiration exhibited a significant increase in the percentage of GD2+Tie2+ cells ^(8)^, which are NP progenitor cells ^(9)^, suggesting that the implanted soft biomaterial induces endogenous NP cells and NP progenitor cells, leading to endogenous IVD repair ^(1), (8)^. Based on these results, a clinical trial was conducted to implant UPAL gel into the disc after discectomy in patients undergoing surgery for lumbar disc herniation ^(10)^. In addition, focusing on the “pain” associated with IVD, the UPAL gel suppressed the expression of inflammatory cytokines and nerve growth factor receptors, demonstrating its usefulness in reducing discogenic back pain (Figure 1, right) ^(11)^.

**Figure 1.**
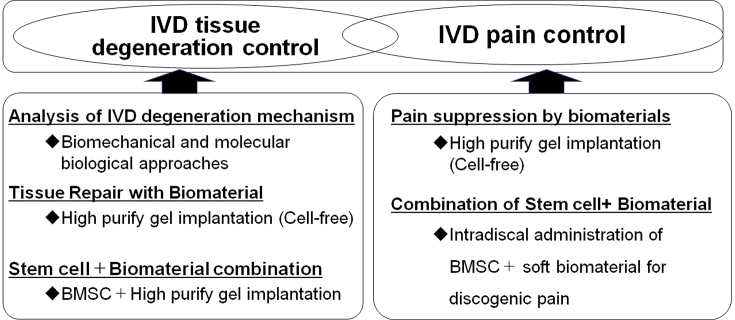
Development approach to the next-generation treatment for intervertebral disc disorders. IVD, intervertebral disc; BMSC, bone marrow-derived mesenchymal stem cell.

However, there are clear limitations to treatment with biomaterials alone in middle-aged and older patients with poor self-repair capacity. Thus, we focused on stem cells as a treatment for IVD regeneration ^(1), (3)^, of which we investigated the use of bone marrow-derived mesenchymal stem cells (BMSCs) ^(12)^. The efficacy of intradiscal administration of BMSCs in patients with chronic low back pain associated with disc degeneration has been reported, and several clinical trials have been conducted ^(1), (2)^. Implantation of a mixture of allogeneic BMSCs and UPAL gel in degenerated IVDs was found to promote tissue repair compared with gel alone in *in vivo* studies (Figure 1, left) ^(12)^. Regarding the mechanism of IVD regeneration by BMSCs and biomaterials, the expression of NP cell markers increased over time in transplanted BMSCs in these studies, indicating the differentiation of transplanted BMSCs into NP cells *in vivo* ^(12)^. Furthermore, high type II collagen-positive cell rates were observed in IVDs transplanted with BMSCs in combination with gel, indicating the enhanced production of extracellular matrix in discs transplanted with allogeneic BMSCs ^(12)^. However, the problems with conventional BMSCs include cell quality degradation during cell culture, difficulty in maintaining cell quality, and the presence of nondifferentiating contaminating cells ^(2), (13)^. Rapidly expanding clones (RECs) are high-quality, highly purified, allogeneic human BMSC cells isolated and extracted directly from the bone marrow using a cell sorter based on the expression of two cell surface markers, namely, CD271 and CD90 ^(13), (14)^. RECs are genetically stable, ultrapurified, Good Manufacturing Practices-compliant clonogenic BMSCs and do not show lot-related differences in clinical applications ^(13), (14)^. Implantation of REC in combination with UPAL gel into the IVD was found to promote tissue repair in a sheep model of severe IVD degeneration compared with gel alone, indicating that UPAL gel prevents cell leakage as a cell carrier and facilitates BMSC activation ^(1), (13)^. A prospective, double-blind, randomized, controlled trial of REC in combination with UPAL gel has also been conducted in middle-aged and older patients with lumbar spinal canal stenosis complicated by disc herniation ^(15)^.

Basic research was conducted from the perspective of a reverse translational approach to elucidate the effects of the combination of biomaterials and BMSCs on pain suppression (Figure 1, right). To develop a novel local injection therapy for discogenic low back pain, the biomaterial was designed to be injected into the IVD in a sol rather than a gel state. Injection of a mixture of REC and UPAL nongelling solution in a rat caudal IVD punch model suppressed IVD degeneration as well as the expressions of inflammatory cytokines and nerve growth factor receptors (TNF-α, IL-6, and TrkA) ^(2)^. Moreover, a mixture of REC and UPAL suppressed nociceptive behavior compared with UPAL alone ^(2)^. This research has the great advantage of being clinically applicable as it used stem cells and soft biomaterials that have been clinically used in humans. The results indicate that the method of a single injection of stem cells and soft biomaterial has a potential application in the treatment of discogenic pain and as a regenerative therapy ^(2)^. This method is minimally invasive and safe, does not require hospitalization or surgery, and could offer greater pain control and disc regeneration than existing treatments for low back pain ^(2)^.

Thus, from the perspective of the reverse translational approach, which aims to return to basic research and elucidate the mechanisms of the issues raised by the results of clinical studies, the development of a new next-generation treatment that suppresses IVD tissue degeneration and is useful in reducing discogenic low back pain is expected.

## Article Information

This article is based on the study, which received the Medical Research Encouragement Prize of The Japan Medical Association in 2023.

### Conflicts of Interest

None

### Author Contributions

Katsuhisa Yamada: Conceptualization, Methodology, Formal analysis, Writing - original draft, Investigation; Hideki Sudo: Conceptualization, Methodology, Formal analysis, Writing - review and editing, Supervision, Project administration; Norimasa Iwasaki: Supervision.
